# SNP discovery and genotyping using Genotyping-by-Sequencing in Pekin ducks

**DOI:** 10.1038/srep36223

**Published:** 2016-11-15

**Authors:** Feng Zhu, Qian-Qian Cui, Zhuo-Cheng Hou

**Affiliations:** 1 National Engineering Laboratory for Animal Breeding and MOA Key Laboratory of Animal Genetics and Breeding, Department of Animal Genetics and Breeding, China Agricultural University, Beijing 100193, China

## Abstract

Genomic selection and genome-wide association studies need thousands to millions of SNPs. However, many non-model species do not have reference chips for detecting variation. Our goal was to develop and validate an inexpensive but effective method for detecting SNP variation. Genotyping by sequencing (GBS) can be a highly efficient strategy for genome-wide SNP detection, as an alternative to microarray chips. Here, we developed a GBS protocol for ducks and tested it to genotype 49 Pekin ducks. A total of 169,209 SNPs were identified from all animals, with a mean of 55,920 SNPs per individual. The average SNP density reached 1156 SNPs/MB. In this study, the first application of GBS to ducks, we demonstrate the power and simplicity of this method. GBS can be used for genetic studies in to provide an effective method for genome-wide SNP discovery.

The domestic duck is an economically important agriculture animal and is consumed worldwide, especially in Asia^1^. Meanwhile, duck is also a suitable material for population genetics and evolutionary studies^2^,^3^. However, there is a limitation for population genetics studies and genomic selection due to the lack of a duck-specific DNA chip platform. Reduced-representation methods using restriction enzymes for the digestion reduce the genome complexity and are suitable for assaying SNPs from large numbers of samples with high reproducibility and low per-sample cost^4^. Genotyping by sequencing (GBS) is one such highly efficient strategy for genome-wide SNP detection and this approach has been successfully applied to aquatic, plants, and animals like chicken, pig and cattle^5^,^6^,^7^,^8^,^9^,^10^,^11^,^12^. In this study, we developed a GBS strategy (Fig. 1) and applied it for SNP detection in the domestic Pekin duck, and evaluated the results by PCR-RFLP. Our theoretic analysis and experimental data showed this is a low cost and effective method for discovering SNPs in animal genomes for which chip microarrays are not yet available.

## Results

### The selection of restriction enzyme

**Figure 2:**
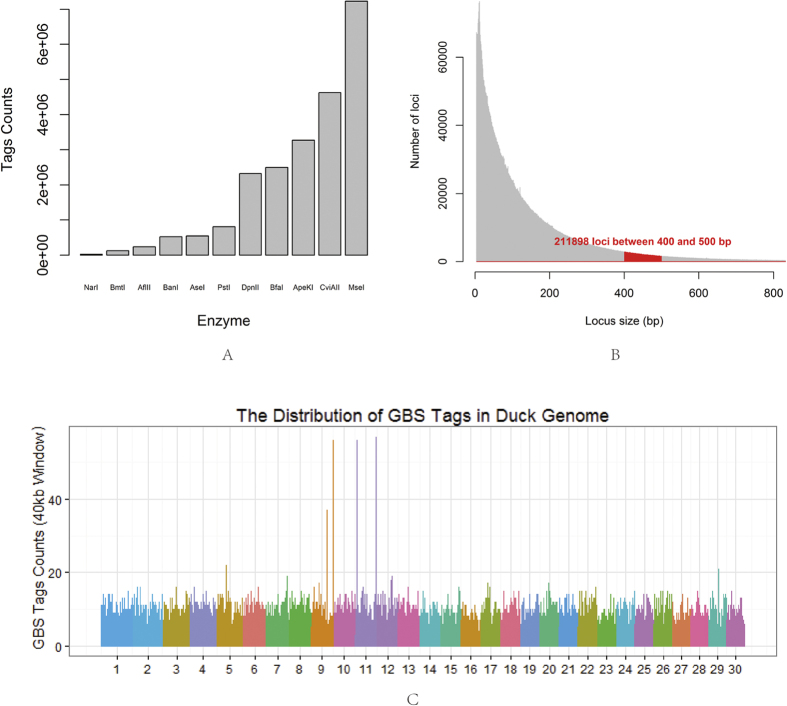
(**A**) Simulation of DNA tags with restriction enzyme digestion. Tag counts for restriction enzyme NarI, BmtI, AflII, BanI, AseI, PstI, DpnII, BfaI, ApeKI, CviAII and MseI were estimated from the duck genome (BGI 1.0), based on loci of restriction enzyme nucleotide sequence recognition. (**B**) Distribution of Tag lengths with MseI digestion. The red area represents the suitable tag size which ranged from 400–500 bp. (**C**) Distribution of sequenced fragments count in 40-kb windows along the 30 longest scaffolds.

We used 11 commonly used restriction enzymes to conduct an *in silico* digestion study. The results of simulated digestion are illustrated in Fig. 2A. Tag number is one important index for evaluating enzyme digestion performance. *Mse*I digestion was predicted to achieve more tags than other enzymes. The smooth tag size distribution curve for *Mse*I supported its choice as a good candidate for this GBS study (Fig. 2B). Aside from the tag number, the genome-wide distribution of tags is another characteristic for enzyme selection. Theoretic results also suggest that *Mse*I is better than other enzymes and has an even distribution pattern across the genome (Fig. 2C, Supplementary Figs S1 and S2). From consideration of tags on repeat regions and degenerative sites, *Mse*I also achieved the highest tag number (Supplementary Table S1). Based on these analyses, *Mse*I is the best candidate enzyme, from the eleven considered, for a GBS study in ducks. Regarding reads length and sequencing depth, 500 bp tag size would be suitable for this study. There are 211,898 tags whose length ranged from 400–500 bp in the *in silico* study (Fig. 2B).

### SNP discovery

**Figure 3:**
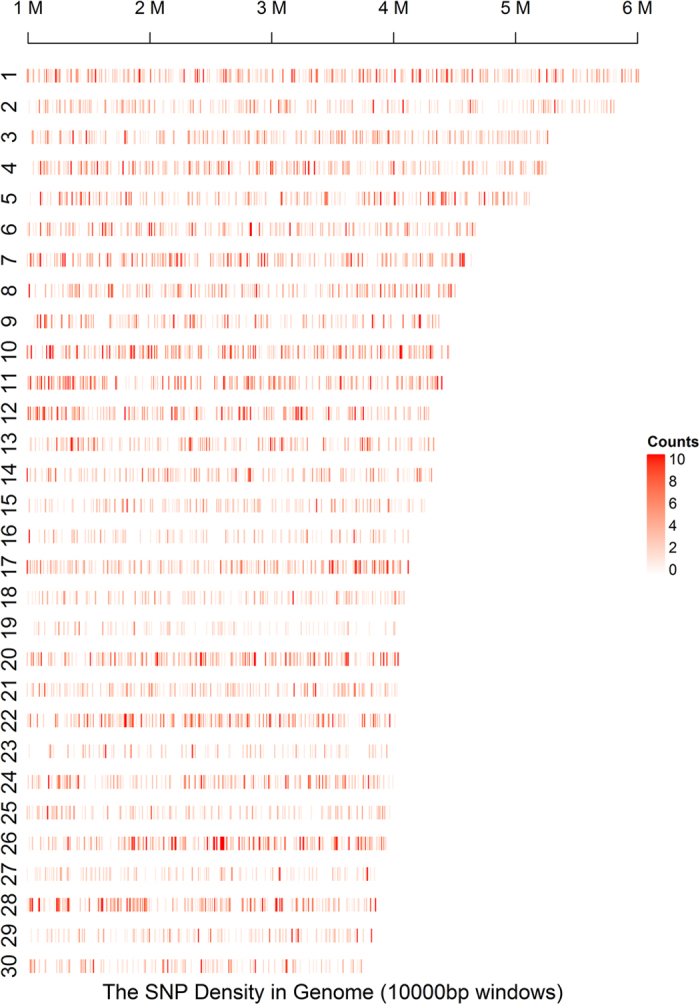
Distribution of SNP counts in 10-kb windows along the genome. Only SNPs on the 30 longest scaffolds are shown. The shade of red scale represents the variation density of SNPs per window.

A total of 544 million clean reads (63.25 Gb) were generated and 96.12% (523 million reads) of these were mapped to the duck genome with an average mapping rate of 96.25% (Supplementary Table S2). In total, about 13% of the genome was covered with tags, compared with 6.9% coverage predicted from *in silico* digestion. In total, 49,413 of GBS fragments were detected; tag length ranged from 39830 to 4230 bp, with median length 465 bp. Individual data information is shown in Supplementary Table S3. A total of 169,209 high-confidence SNPs were retained from all samples, with a mean of 55,920 SNPs identified for each individual (40,897 to 63,927) (Supplementary Table S4). Among the called genotypes, the number of SNPs was counted at 10-kb window size along the pseudo-chromosome displayed in Fig. 3. The SNP density reached 1156 SNPs/Mb, with an average of 41.23 SNPs identified for each fragment. The mean of the SNP missing rate was 5.64% and only 4 samples had a missing rate greater than 20% (Supplementary Table S4).

### SNP validation

To validate results, we performed an *in silico* study with 50 randomly selected SNPs. After considering chromosomal distribution of SNPs and suitable enzyme digestion loci, we chose 24 SNPs to perform PCR-RFLP analysis. These SNPs were randomly distributed in the duck genome with approximately one selected site per chromosome. The results of PCR-RFLP assay are illustrated in Supplementary Table S5. A total of 982 sites were identified in PCR-RFLP and 280 SNPs were found, of which 90% (251/280) of identified SNPs were concordant with GBS’s results. Moreover, 94% (921/982) of genotypes were consistent with the GBS results. All the SNPs found using GBS were successfully validated by PCR-RFLP. Although randomicity and incomplete sequence coverage could lead to inconsistent results between GBS and PCR-RFLP, the PCR-RFLP assay results showed that the SNP library obtained by GBS were highly credible.

## Discussion

Genomic selection and genome-wide association studies need 10^5^ to 10^6^ SNPs. However, many non-model species do not have reference chips for detecting variation. To solve this issue, one option is to generate more sequencing data as sequencing costs continue to fall quickly. The GBS method has great potential for application to the genomes of agricultural animals, as an alternatives to chip platforms. Pertille and coworkers sequenced 462 chicken using GBS method and identified 67,096 SNP with a 4.66% coverage of whole genome^11^. A pig sequencing experiment detected putative SNPs with an average density of 0.33 SNPs/10 Kb^9^. Additionally 63,797 SNPs were identified in a cattle study^12^. In this study, about 0.5-1X genome coverage data were obtained and the SNP density was found to be 1156 SNPs/Mb. Compared to other agricultural animals, our results showed excellent performance and high coverage for digested tags in ducks. The performance was slightly lower than that of the chicken study due to the population size of our study and the quality of the genome. A few individuals with relatively high missing rate (>20%) can be rescued using imputation methods when the sample size is large^13^,^14^, especially in the designing genomic selection study.

We observed that the ratio of genome coverage was a mean of 13%, higher than the 6.9%, predicted coverage. Two possibilities might lead to this result. Firstly, the duck reference genome still has many gaps. In the current duck reference genome, there are more than 70,000 contigs/scaffold. Therefore, the real number of predicted fragments cannot represent the real data until the quality of the reference genome is improved. Another reason is that shorter fragments were used in sequencing, even though a narrow selection range was set using a Pippin system. In practice, higher genome coverage will obtain more SNPs, but with reduced sequencing depth in some loci with the same sequencing data. In summary, we genotyped 49 Pekin ducks using GBS and identified 169,209 confident SNPs. We have demonstrated that GBS is a highly effective method for accessible and low cost genome-wide genotyping.

## Methods

### Ethics statement

All experiments were performed according to regulations and guidelines established by the Animal Care and Use Committee of China Agricultural University (permit number: DK996). All protocols and procedures were approved by the Beijing Administration Committee of Laboratory Animals under the leadership of the Beijing Association for Science and Technology (permit number: SYXK 2007–0023). Blood was extracted from the wing-vein via vacuum piping, with 75% alcohol/cotton ball for disinfection. All efforts were made to minimize animal suffering during the study.

### Samples preparation

Forty-nine Pekin ducks from the same flock were randomly selected at the Beijing Jinxing Golden Star Duck Centre. Birds were fed *ad libitum* from 0 to 6 weeks. A blood sample was collected from each individual.

### DNA extract, library construction, Sequencing

Genomics DNA was extracted from blood using the standard phenol/chloroform method. An *in silico* digestion of the duck reference genome (BGI 1.0, Ensemble 82) was performed to choose the appropriate restriction endonuclease using R package SimRAD^15^. According to the results of simulated digestion, 100 ug genomic DNA was digested with restriction endonuclease *Mse*I, which recognizes a 4-bp sequence (TTAA) and creates a 2-bp overhang (Supplementary Table S6). Then a set of variable barcode adapters that recognize Mse1-compatible sequences were ligated to the digested DNA fragments. The ligation mixture was purified using Ampure XP beads. Fragments ranging from 550 to 580 bp, including adapter sequences, were purified with gel extraction. Next, the restriction fragments were enriched by PCR amplification with adapter-specific primers. The quality evaluation was performed by ABI StepOne Plus. The data of 2 × 125 bp pair-end reads were generated by the Illumina HiSeq2500.

### Mapping

The raw reads that had <20 sequence quality score and <50-bp of sequence length were removed, and then barcode sequences were eliminated. The clean sequences were aligned to the duck reference genome (BGI 1.0, Ensemble 82) using Burrows-Wheeler Aligner (BWA) with the default parameters^16^. Read grouping and removal of PCR duplicates were done using Picard (http://picard.sourceforge.net.). The data were deposited in the NCBI sequence read archive (SRP068685).

### Mutation detection

The genome analysis toolkit (GATK) was used to perform local realignment of reads to correct misalignments, and then to detect the SNPs and call the genotypes (*-stand_call_conf 20 -stand_emit_conf 20, other parameters were default*)^17^. Two criteria were used to identify the SNPs: 1. the missing rate of each locus could not be more than 0.2; 2. the mapping depth of each locus per sample should be more than 4. The information of tags calling si illustrated in Supplementary Table S3

### PCR-RFLP

Restriction enzymes for the PCR-RFLP assay were selected using information from REBASE (http://rebase.neb.com)^18^. Primers for PCR were designed using Primer-Blast (http://www.ncbi.nlm.nih.gov/tools/primer-blast/)^19^. Conditions for the PCR were as follows: 94 °C for 5 min; 34 cycles of 94 °C for 30 s, 60–62 °C for 30 s and 72 °C for 1 min. This wasfollowed by a further 10 min extension at 72 °C. The restriction assay was performed at 37 °C for 2 h.

## Additional Information

**How to cite this article**: Zhu, F. *et al.* SNP discovery and genotyping using Genotyping-by-Sequencing in Pekin ducks. *Sci. Rep.* **6**, 36223; doi: 10.1038/srep36223 (2016).

**Publisher’s note**: Springer Nature remains neutral with regard to jurisdictional claims in published maps and institutional affiliations.

## Supplementary Material

Supplementary Information

## Figures and Tables

**Figure 1:**
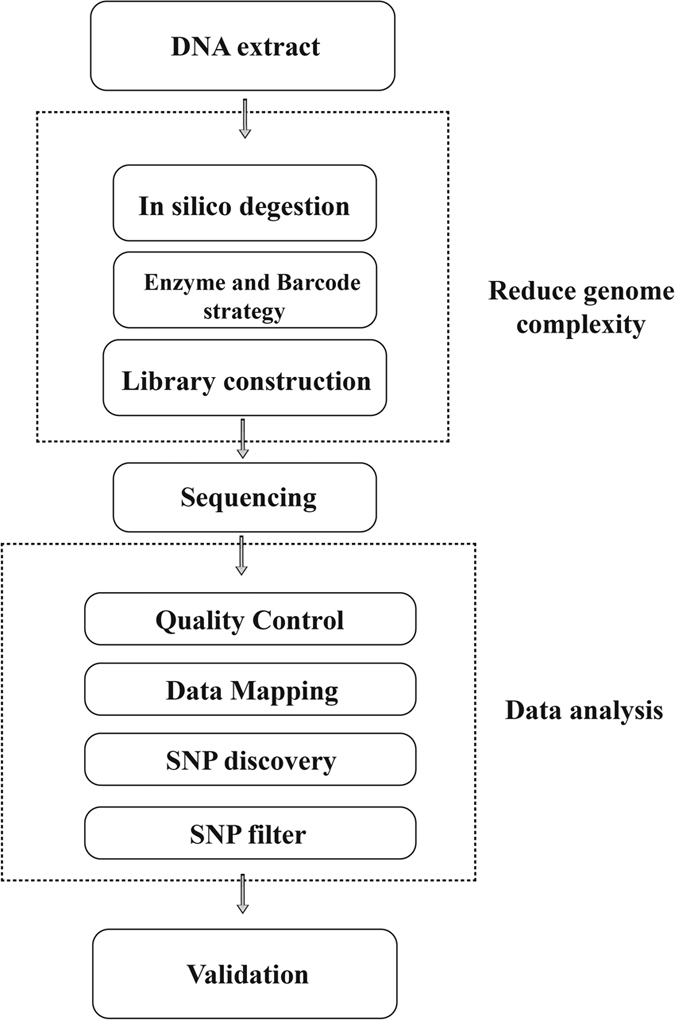
The GBS strategy pipeline. The procedure mainly included three parts: 1. Genome-represent-reduced sequencing; 2. SNP discovery; 3. SNP validation.
