# Supplementary material for: SNP discovery and genotyping using Genotyping-by-Sequencing in Pekin ducks
Source: Sci Rep. 2016 Nov 15;6:36223. doi: 10.1038/srep36223 (PMC5109183; doi:10.1038/srep36223)
Supplement: Supplementary Information [file srep36223-s1.pdf]

SNP discovery and genotyping using Genotyping-by-Sequencing in Pekin ducks

Authors

Feng Zhu[1] , Qian-Qian Cui[1], Zhuo-Cheng Hou[1*]

Supplementary Table

Supplementary Figure

Table S1. Summary for degenerate sites of enzyme reorganization sequences and repeat elements of tags fragment

	Tags Count in Repeat Region	Bases(bp)	Coverage on Repeat Region(%)	Degenerate Sites	Valid Total Counts	Valid Ratio
*ApeK*I	56783	4939070	6.37%	Yes	3273150	98%
*CviA*II	137940	8300198	11.08%	No	4623450	97%
*Mse*I	72447	7216120	7.80%	No	7228565	99%
*Pst*I	10324	793102	1.21%	No	806730	99%
*Nar*I	50	3069	0.01%	No	25622	100%
*Dpn*II	67794	5826996	6.97%	No	2328385	97%
*Bmt*I	404	35332	0.05%	No	129355	100%
*Bfa*I	87337	6299838	7.71%	No	2501245	97%
*Ban*I	5512	567565	0.60%	Yes	525898	99%
*Ase*I	7874	495050	0.92%	No	547575	99%
*Afl*II	1703	122160	0.19%	No	239100	99%

Table S2. Mapping information for Samples (QC)

#Sample	LEN	Clean reads（R1+R2）	Clean bases(bp)	Mapped reads	Mapped bases(bp)	Mapping rate	Mismatch bases(bp)	Mismatch rate
36547	118	4373102	1032052072	8446155	996646290	96.57%	7577331	0.76%
36553	118	7247970	1710520920	13970165	1648479470	96.37%	14089459	0.85%
36555	117	4514457	1056382938	8714753	1019626101	96.52%	7789141	0.76%
36558	117	3547059	830011806	6843449	800683533	96.47%	6682959	0.83%
36560	116	4798534	1113259888	9246445	1072587620	96.35%	9663859	0.90%
36567	116	4429104	1027552128	8542621	990944036	96.44%	7848690	0.79%
36568	115	6607963	1519831490	12728101	1463731615	96.31%	12399886	0.85%
36569	115	5776316	1328552680	11136529	1280700835	96.40%	10839342	0.85%
36593	117	4258853	996571602	8234700	963459900	96.68%	7245950	0.75%
36607	118	3188020	752372720	6134157	723830526	96.21%	6820201	0.94%
36621	117	3102628	726014952	5989379	700757343	96.52%	6645415	0.95%
36634	117	3755367	878755878	7241909	847303353	96.42%	8109751	0.96%
36635	116	5434597	1260826504	10475544	1215163104	96.38%	12672399	1.04%
36648	116	9054794	2100712208	17458197	2025150852	96.40%	17645827	0.87%
36664	116	5598553	1298864296	10798824	1252663584	96.44%	10034667	0.80%
36665	115	8415586	1935584780	16214297	1864644155	96.33%	15365233	0.82%
36674	115	1.1E+07	2599135240	21763451	2502796865	96.29%	21735676	0.87%
36675	118	8959513	2114445068	17268690	2037705420	96.37%	15159857	0.74%
36774	117	5661635	1324822590	10906677	1276081209	96.32%	9878348	0.77%
36775	117	4272960	999872640	8206478	960157926	96.03%	7723019	0.80%
36776	116	6165685	1430438920	11839617	1373395572	96.01%	11890122	0.87%
36784	116	4399913	1020779816	8465090	981950440	96.20%	7718417	0.79%
36798	115	7642603	1757798690	14648327	1684557605	95.83%	14707208	0.87%
36816	115	7696585	1770214550	14782732	1700014180	96.03%	14532224	0.85%
36820	118	3754083	885963588	7220836	852058648	96.17%	6692852	0.79%
36821	117	4086832	956318688	7868810	920650770	96.27%	7288113	0.79%
36822	117	3690042	863469828	7094520	830058840	96.13%	6581397	0.79%
36829	116	6610505	1533637160	12712077	1474600932	96.15%	12563438	0.85%
36837	116	4591589	1065248648	8831811	1024490076	96.17%	8335377	0.81%
36921	115	9319787	2143551010	17948351	2064060365	96.29%	17102581	0.83%
36923	115	3942793	906842390	7584053	872166095	96.18%	7420250	0.85%
36937	118	4874143	1150297748	9428631	1112578458	96.72%	8028554	0.72%
36944	117	4093575	957896550	7888861	922996737	96.36%	7322086	0.79%
36946	117	3012906	705020004	5812869	680105673	96.47%	5406290	0.79%
36954	116	5429424	1259626368	10431876	1210097616	96.07%	10240479	0.85%
36973	116	3306472	767101504	6371087	739046092	96.34%	5778865	0.78%
36986	115	6444391	1482209930	12369585	1422502275	95.97%	11678787	0.82%
36991	115	6030887	1387104010	11591576	1333031240	96.10%	11008242	0.83%
37227	117	7997033	1871305722	15361610	1797308370	96.05%	16141438	0.90%
37238	116	4777566	1108395312	9198741	1067053956	96.27%	9986915	0.94%
37239	116	5478700	1271058400	10492348	1217112368	95.76%	10847786	0.89%

37244	115	4777295	1098777850	9194173	1057329895	96.23%	9421803	0.89%
37248	115	3995298	918918540	7691431	884514565	96.26%	7917576	0.90%
37257	118	5985421	1412559356	11500985	1357116230	96.07%	11578391	0.85%
37264	117	3151495	737449830	6067125	709853625	96.26%	5886264	0.83%
37265	117	5294617	1238940378	10186837	1191859929	96.20%	9812279	0.82%
37270	116	3577239	829919448	6860758	795847928	95.89%	7073856	0.89%
37274	115	8058290	1853406700	15468346	1778859790	95.98%	15564315	0.87%
37283	115	6233690	1433748700	11975738	1377209870	96.06%	11255716	0.82%

Table S3. Summary of missing rate for individual

Sample ID	Missing SNP	Missing Rate
36923	41901	0.247629
36822	37693	0.22276
36821	36428	0.215284
36837	34277	0.202572
36820	33595	0.198541
36555	25919	0.153177
36829	25444	0.15037
36593	22895	0.135306
36921	22469	0.132788
36607	13615	0.0804626
36621	11339	0.0670118
37248	8732	0.0516048
36664	8712	0.0514866
36784	7278	0.0430119
36634	7177	0.042415
36553	6981	0.0412567
37238	6922	0.040908
36547	6829	0.0403584
36775	6580	0.0388868
37244	6190	0.036582
37264	5624	0.033237
36946	5455	0.0322382
37239	5230	0.0309085
37270	5034	0.0297502
36937	4837	0.0285859
37227	4747	0.0280541
36665	4444	0.0262634
36774	4285	0.0253237
36648	3998	0.0236276
36973	3631	0.0214587
36558	3549	0.0209741
36635	3493	0.0206431
36944	3335	0.0197094
36776	3257	0.0192484
36798	3086	0.0182378
36816	3019	0.0178418
36674	2571	0.0151942
36567	2515	0.0148633
37283	2189	0.0129367

36560	1942	0.0114769
36675	1940	0.0114651
36986	1718	0.0101531
36568	1708	0.010094
37274	1663	0.00982808
37265	1610	0.00951486
36954	1593	0.00941439
36569	1544	0.00912481
36991	1443	0.00852792
37257	1166	0.00689089

Table S4. Summary of variant position heterozygosity for individuals

Sample ID	Total	Ref	Alt	Hete	Homo	Homo rate(%)	Hete rate(%)
36547	147077	94203	52874	29022	23852	16.22	19.73
36553	160763	102074	58689	30793	27896	17.35	19.15
36555	138144	92663	45481	16807	28674	20.76	12.17
36558	157487	97511	59976	35030	24946	15.84	22.24
36560	164405	102804	61601	35516	26085	15.87	21.6
36567	160805	101668	59137	33556	25581	15.91	20.87
36568	166705	103572	63133	37179	25954	15.57	22.3
36569	166568	103171	63397	37662	25735	15.45	22.61
36593	141287	93609	47678	18935	28743	20.34	13.4
36607	130672	83752	46920	25711	21209	16.23	19.68
36621	135246	87250	47996	24979	23017	17.02	18.47
36634	145775	92690	53085	29854	23231	15.94	20.48
36635	159116	98786	60330	36651	23679	14.88	23.03
36648	164056	104174	59882	33060	26822	16.35	20.15
36664	157237	103303	53934	23851	30083	19.13	15.17
36665	163443	104015	59428	31268	28160	17.23	19.13
36674	166107	105359	60748	32726	28022	16.87	19.7
36675	166513	105319	61194	33531	27663	16.61	20.14
36774	162578	102285	60293	32762	27531	16.93	20.15
36775	158494	100451	58043	30580	27463	17.33	19.29
36776	163782	103506	60276	31561	28715	17.53	19.27
36784	157500	101840	55660	26306	29354	18.64	16.7
36798	164546	104898	59648	30857	28791	17.5	18.75
36816	164836	101985	62851	36897	25954	15.75	22.38
36820	127961	83420	44541	19960	24581	19.21	15.6
36821	124160	82546	41614	17128	24486	19.72	13.8
36822	122104	79939	42165	18985	23180	18.98	15.55
36829	135346	86855	48491	23030	25461	18.81	17.02
36837	124743	81261	43482	20558	22924	18.38	16.48
36921	139516	90003	49513	25325	24188	17.34	18.15
36923	118683	77786	40897	17383	23514	19.81	14.65
36937	155369	97866	57503	32498	25005	16.09	20.92
36944	159468	99872	59596	33676	25920	16.25	21.12
36946	152943	96764	56179	30742	25437	16.63	20.1
36954	166194	102816	63378	38684	24694	14.86	23.28
36973	158078	99492	58586	33654	24932	15.77	21.29
36986	166792	102865	63927	38759	25168	15.09	23.24
36991	167002	103440	63562	36575	26987	16.16	21.9
37227	156827	98327	58500	33308	25192	16.06	21.24
37238	151908	96073	55835	31305	24530	16.15	20.61

37239	155231	96722	58509	33381	25128	16.19	21.5
37244	153965	96819	57146	31652	25494	16.56	20.56
37248	148759	97865	50894	22640	28254	18.99	15.22
37257	166809	102946	63863	37811	26052	15.62	22.67
37264	151975	96342	55633	30150	25483	16.77	19.84
37265	165077	101411	63666	37800	25866	15.67	22.9
37270	154468	96959	57509	31522	25987	16.82	20.41
37274	165437	104020	61417	34192	27225	16.46	20.67
37283	162960	103834	59126	32090	27036	16.59	19.69

Table S5. Results of PCR-RFLP analysis

Chromosome	Position	REF	ALT	Restriction Enzyme		36547	36553	36555	36558	36560	36567	36568	36569	36593	36607	36621	36634	36635	36648	36664	36665
KB742444.1	590170	A	C	HindIII	GBS	1	0	1	0	0	2	1	0	x	2	1	0	0	1	0	1
					PCR-RFLP	1	0	1	0	0	2	1	0	0	2	1	0	0	1	0	1
KB743090.1	1125934	C	T	HindIII	GBS	0	0	x	1	1	1	0	0	2	0	0	0	0	0	0	0
					PCR-RFLP	0	0	0	1	1	1	0	0	1	1	0	0	1	0	0	0
KB743525.1	1034355	A	G	HindIII	GBS	0	0	x	0	0	0	0	1	0	0	0	0	1	0	1	0
					PCR-RFLP	0	0	1	0	0	0	0	1	0	0	0	0	1	0	1	0
KB744292.1	182237	C	G	HindIII	GBS	0	x	0	0	0	0	0	0	x	0	x	0	1	0	0	0
					PCR-RFLP	0	0	0	0	0	0	0	0	0	0	1	0	1	0	0	0
KB749455.1	105326	G	A	HindIII	GBS	0	1	1	1	0	0	1	0	0	1	0	0	1	1	0	1
					PCR-RFLP	0	1	1	1	0	0	1	0	2	1	0	0	1	1	0	1
KB743520.1	105729	T	A	BglII	GBS	0	0	2	0	0	0	0	0	0	0	0	0	2	0	0	1
					PCR-RFLP	1	1	0	0	0	1	0	0	0	0	0	1	1	0	0	1
KB743176.1	539915	T	A	BglII	GBS	0	0	0	0	0	0	0	0	0	0	0	0	0	1	0	0
					PCR-RFLP	0	0	0	0	0	0	0	0	0	0	0	0	0	1	0	0
KB744001.1	190730	C	A	BglII	GBS	2	0	0	0	0	2	0	0	0	2	0	0	2	0	0	0
					PCR-RFLP	x	0	0	0	0	2	0	0	0	2	0	0	2	0	0	0
KB744509.1	57616	G	T	BglII	GBS	0	0	0	0	0	0	0	0	0	0	0	0	0	0	0	0
					PCR-RFLP	0	0	0	0	x	0	0	x	0	0	0	0	0	0	0	0
KB742513.1	541077	T	C	NdeI	GBS	0	0	0	1	0	1	0	1	x	0	1	1	1	1	0	0
					PCR-RFLP	0	0	1	1	0	1	0	1	1	0	1	1	1	1	0	0
KB742580.1	1315947	T	C	NdeI	GBS	0	0	0	0	0	0	0	0	0	0	0	0	0	0	0	1
					PCR-RFLP	0	0	0	0	0	0	0	0	0	0	0	0	0	0	0	1
KB743520.1	105729	T	A	NdeI	GBS	2	0	0	0	1	1	2	0	x	0	0	0	0	2	0	0

					**PCR-RFLP**	**1**	**0**	**0**	**0**	**1**	**1**	**2**	**0**	**0**	**0**	**0**	**0**	**0**	**2**	**0**	**0**
**KB743520.1**	**105729**	**T**	**A**	**NdeI**	**GBS**	**1**	**x**	**0**	**0**	**2**	**2**	**2**	**0**	**x**	**0**	**0**	**2**	**1**	**1**	**x**	**2**
					**PCR-RFLP**	**1**	**1**	**0**	**0**	**2**	**2**	**2**	**0**	**2**	**0**	**0**	**2**	**1**	**1**	**1**	**2**
**KB743157.1**	**1557416**	**A**	**G**	**NdeI**	**GBS**	**0**	**0**	**0**	**0**	**0**	**0**	**0**	**0**	**0**	**0**	**0**	**2**	**0**	**0**	**0**	**0**
					**PCR-RFLP**	**0**	**0**	**0**	**0**	**0**	**0**	**0**	**0**	**0**	**0**	**0**	**2**	**0**	**0**	**0**	**0**
**KB743136.1**	**769513**	**A**	**C**	**NdeI**	**GBS**	**2**	**0**	**2**	**1**	**2**	**1**	**2**	**2**	**2**	**1**	**2**	**2**	**1**	**2**	**2**	**2**
					**PCR-RFLP**	**2**	**0**	**2**	**1**	**2**	**1**	**2**	**2**	**2**	**1**	**2**	**2**	**1**	**2**	**2**	**2**
**KB743177.1**	**144500**	**C**	**A**	**EcoRI**	**GBS**	**0**	**0**	**0**	**0**	**0**	**0**	**0**	**0**	**0**	**0**	**0**	**0**	**0**	**0**	**0**	**0**
					**PCR-RFLP**	**0**	**0**	**0**	**0**	**0**	**0**	**0**	**0**	**0**	**0**	**0**	**0**	**0**	**0**	**0**	**0**
**KB742992.1**	**1628917**	**C**	**T**	**EcoRI**	**GBS**	**0**	**0**	**x**	**0**	**0**	**0**	**0**	**0**	**0**	**0**	**0**	**1**	**0**	**0**	**0**	**0**
					**PCR-RFLP**	**0**	**0**	**0**	**0**	**0**	**1**	**1**	**0**	**0**	**0**	**0**	**1**	**0**	**0**	**0**	**0**
**KB742605.1**	**1814948**	**A**	**G**	**EcoRI**	**GBS**	**0**	**0**	**x**	**0**	**0**	**0**	**0**	**0**	**x**	**0**	**x**	**0**	**0**	**0**	**0**	**0**
					**PCR-RFLP**	**0**	**0**	**1**	**0**	**0**	**0**	**0**	**0**	**0**	**0**	**1**	**0**	**0**	**0**	**0**	**0**
**KB743111.1**	**1149319**	**A**	**T**	**XbaI**	**GBS**	**0**	**x**	**x**	**0**	**0**	**0**	**0**	**0**	**x**	**0**	**0**	**0**	**0**	**0**	**x**	**0**
					**PCR-RFLP**	**0**	**x**	**0**	**0**	**0**	**0**	**0**	**0**	**0**	**0**	**0**	**0**	**0**	**0**	**x**	**0**
**KB742622.1**	**1524460**	**T**	**C**	**XbaI**	**GBS**	**0**	**0**	**1**	**0**	**0**	**0**	**0**	**0**	**0**	**0**	**0**	**0**	**0**	**0**	**0**	**0**
					**PCR-RFLP**	**0**	**0**	**1**	**0**	**0**	**0**	**0**	**0**	**0**	**0**	**0**	**0**	**0**	**0**	**0**	**0**
**KB743226.1**	**797005**	**G**	**A**	**XbaI**	**GBS**	**0**	**0**	**0**	**1**	**0**	**1**	**0**	**1**	**1**	**0**	**0**	**0**	**1**	**0**	**0**	**0**
					**PCR-RFLP**	**0**	**0**	**1**	**1**	**0**	**1**	**0**	**1**	**1**	**0**	**0**	**0**	**1**	**0**	**0**	**0**
**KB743608.1**	**1135493**	**C**	**T**	**XbaI**	**GBS**	**x**	**0**	**2**	**2**	**1**	**2**	**1**	**1**	**0**	**0**	**x**	**0**	**1**	**1**	**2**	**1**
					**PCR-RFLP**	**2**	**0**	**2**	**2**	**1**	**2**	**1**	**1**	**0**	**0**	**2**	**0**	**1**	**1**	**2**	**1**

The genotype values are 0 for the reference genotype, 1 for the heterozygosity, 2 for the altered genotype. x denotes as the unclear genotype.

36674	36675	36774	36775	36776	36784	36798	36816	36820	36821	36822	36829	36837	36923	36937	36944	36954	36973	36986	36991	37227	37238	37239	37244
1	1	2	0	1	2	2	0	2	0	2	0	2	x	1	0	1	1	1	1	1	1	1	1
1	1	2	0	1	2	2	0	2	0	2	0	2	1	1	0	1	1	1	1	1	1	1	1
1	0	1	0	0	0	0	0	0	0	0	0	x	0	0	0	1	0	0	1	0	0	2	0
1	0	1	1	0	0	0	0	0	0	0	0	0	0	0	0	1	0	0	1	0	0	2	0
x	2	0	0	x	x	1	1	x	0	0	0	1	x	0	0	1	0	0	1	1	0	1	1
0	2	0	0	0	1	1	0	0	0	1	0	1	1	0	0	1	0	0	1	1	0	1	1
1	1	0	0	0	0	0	0	0	1	x	0	0	1	0	0	0	0	0	1	0	0	2	1
1	1	x	0	0	0	0	1	0	1	0	x	0	1	x	0	0	0	0	1	0	0	2	1
0	0	0	2	0	1	0	0	0	1	1	1	1	0	1	1	1	0	0	0	1	0	1	1
0	0	0	2	0	1	0	1	0	1	1	1	1	0	1	1	1	0	0	0	1	0	1	1
0	0	0	0	0	0	0	1	0	0	1	0	0	0	0	0	0	0	1	0	2	0	0	0
0	0	0	0	0	0	0	1	0	0	1	0	0	0	1	0	0	0	1	0	1	0	0	0
0	0	0	0	0	0	0	0	0	x	0	0	x	x	0	0	0	0	0	0	0	0	0	0
0	0	0	0	0	0	0	0	0	0	0	0	0	1	0	0	0	0	0	0	0	0	0	0
0	0	0	0	0	0	0	0	0	0	0	0	1	x	0	0	0	0	0	0	2	2	0	2
0	0	0	0	0	0	0	0	0	0	0	0	1	0	0	0	0	0	0	0	2	2	0	2
0	0	x	0	0	1	0	0	x	0	x	x	0	0	0	0	0	0	0	0	0	0	0	0
0	0	0	0	0	1	0	0	0	0	0	0	0	0	0	0	0	0	0	0	0	0	0	0
2	1	2	x	0	0	2	0	1	x	x	1	0	x	1	x	1	0	1	0	1	2	0	0
2	1	1	0	0	0	2	0	1	1	1	1	0	0	1	1	1	0	1	0	1	2	0	0
0	0	0	0	0	0	0	0	0	0	0	0	0	0	0	0	0	0	0	0	0	0	0	0
0	0	0	0	0	0	0	0	0	0	0	0	0	0	0	0	0	0	0	0	0	0	0	0
0	2	2	0	1	0	1	0	1	0	1	0	1	1	0	1	0	0	0	0	0	0	x	x
0	2	2	0	1	0	1	0	1	0	1	0	1	1	0	1	0	0	0	0	1	0	0	1

1	2	2	1	0	2	2	2	2	2	1	1	1	2	1	1	2	2	2	2	0	x	0	0
1	2	2	1	0	2	2	2	1	1	1	1	1	2	1	1	2	2	2	2	1	2	1	1
0	0	0	0	2	0	1	0	0	0	0	0	0	0	0	0	0	0	0	0	0	x	0	0
0	0	0	0	2	0	1	0	0	0	0	0	0	0	0	0	0	0	0	0	0	0	0	0
1	2	2	1	1	0	2	2	2	2	2	2	2	x	1	1	1	2	2	1	1	2	1	2
1	2	2	1	1	2	2	2	2	2	2	2	2	2	1	1	1	2	2	1	1	2	1	2
0	0	0	0	0	0	0	0	1	0	0	0	0	0	0	0	0	0	0	0	0	0	0	0
0	0	0	0	0	0	0	0	0	0	0	0	0	0	0	0	0	0	0	0	0	0	0	0
0	0	0	0	0	0	0	0	0	0	0	0	0	2	0	0	0	0	0	0	0	0	0	0
0	0	0	0	0	0	0	0	0	0	0	0	0	2	0	0	0	0	0	0	0	0	0	0
0	0	0	0	0	0	0	0	0	0	0	0	0	0	0	0	0	0	1	0	0	0	0	0
0	0	1	0	0	0	0	0	0	0	0	0	0	0	0	0	0	0	1	0	0	0	0	0
0	0	0	0	0	0	0	0	0	0	0	0	0	0	2	0	0	0	0	0	0	0	0	0
0	0	0	0	0	0	0	0	0	0	0	0	0	0	1	0	0	0	0	0	0	0	0	0
0	0	1	0	0	0	0	0	0	x	2	0	0	0	0	0	0	0	0	0	0	0	0	0
0	0	1	0	0	0	0	0	0	0	1	0	0	0	1	0	0	0	0	0	0	1	0	0
1	0	0	1	0	1	1	0	x	0	1	0	0	0	1	1	0	0	0	2	0	0	1	1
1	0	0	1	0	1	1	0	1	0	1	0	0	1	1	1	0	0	0	2	0	0	1	1
0	2	2	2	1	1	2	1	0	2	1	1	2	2	2	0	2	1	1	1	0	0	1	2
0	2	2	2	1	1	2	2	0	2	1	1	2	2	2	1	2	1	1	1	0	0	1	2

37248	37257	37264	37265	37270	37274	37283
2	1	1	2	0	1	0
2	1	1	2	0	1	0
0	1	0	0	0	1	0
0	1	0	0	0	1	0
0	0	2	0	0	0	0
0	0	2	0	0	0	0
x	0	1	0	0	0	0
1	x	x	1	0	x	0
0	1	0	1	1	0	1
0	1	1	1	1	0	1
0	0	0	0	1	0	0
0	0	1	0	0	0	0
0	0	0	0	0	0	0
0	0	0	0	0	0	0
0	0	0	0	0	0	0
0	x	x	0	0	0	0
0	0	0	0	0	0	0
0	0	0	0	1	0	0
0	0	0	0	2	1	1
0	0	0	0	1	1	1
0	0	0	0	1	0	0
0	0	0	0	1	0	0
0	0	1	1	0	0	1
1	0	1	1	0	0	1

2	2	1	1	2	2	1
2	2	1	1	2	0	1
0	0	0	0	0	1	1
0	0	0	0	0	1	1
2	1	2	1	x	2	2
2	1	2	1	1	2	2
0	0	0	0	0	0	0
0	0	0	0	0	0	0
0	0	0	0	0	0	0
0	0	0	0	0	1	0
0	0	0	0	0	0	0
1	0	0	0	0	0	0
x	0	0	0	0	0	0
0	0	0	0	0	0	0
0	0	0	0	0	0	0
0	1	0	0	0	0	0
0	1	0	0	0	1	1
1	1	0	0	0	x	1
1	0	0	1	1	1	1
1	0	0	1	1	1	1

Table S6. Barcode information

		Positions											
		1	2	3	4	5	6	7	8	9	10	11	12
Barcode	1	A	A	G	C	T	A	A					
	2	T	A	A	G	T	A	A					
	3	T	C	T	C	C	T	A	A				
	4	C	T	G	A	C	T	A	A				
	5	A	G	C	A	G	G	T	A	A			
	6	C	T	C	C	A	G	T	A	A			
	7	T	T	C	T	A	G	G	T	A	A		
	8	T	G	A	T	A	A	C	T	A	A		
	9	G	T	A	A	G	C	C	G	T	A	A	
	10	G	A	T	C	G	T	C	G	C	T	A	A
	11	C	C	G	T	C	C	G	C	G	T	A	A
	12	A	C	T	G	G	A	C	C	G	T	A	A
Rate	A	25%	25%	25%	25%	25%	33%	33%	38%	42%	38%	50%	44%
	T	33%	33%	25%	25%	17%	25%	17%	21%	17%	38%	17%	19%
	C	25%	25%	25%	33%	33%	17%	33%	21%	17%	13%	17%	19%
	G	17%	17%	25%	17%	25%	25%	17%	21%	25%	13%	17%	19%

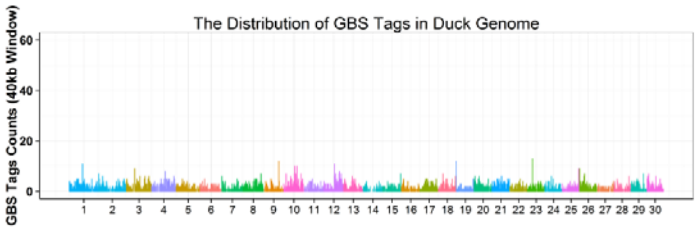

***Pst*I**

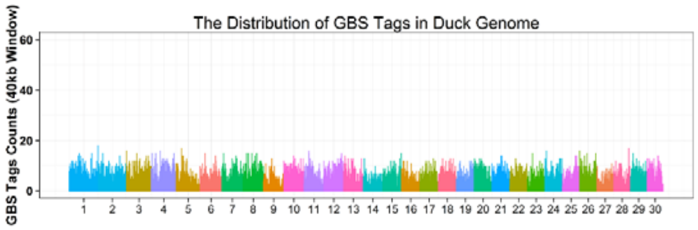

***ApeK*I**

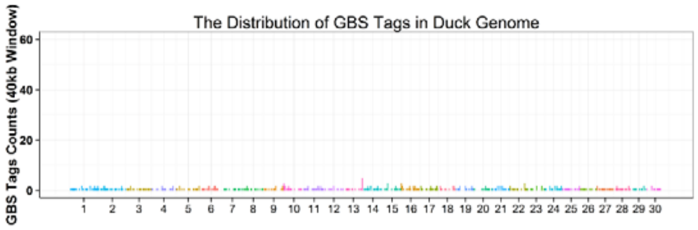

***Afl*II**

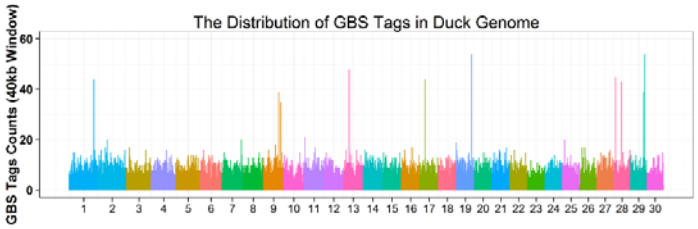

***Bfa*I**

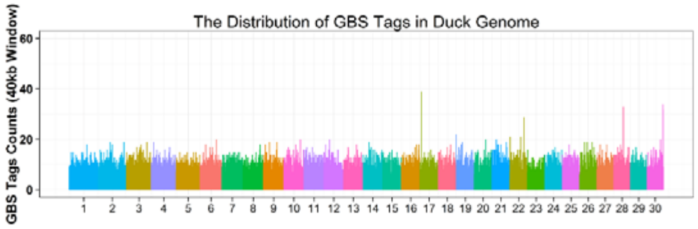

***CviA*II**

Figure S1. Distribution of silico digested fragments count in 40-kb windows along the longest of the first thirty persudo-chromosome. Genomic sequences were distinguished by color.

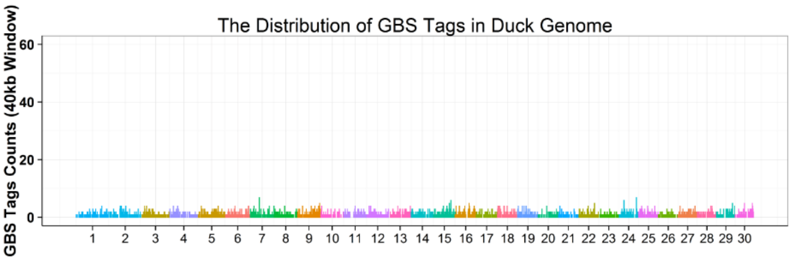

***Ase*I**

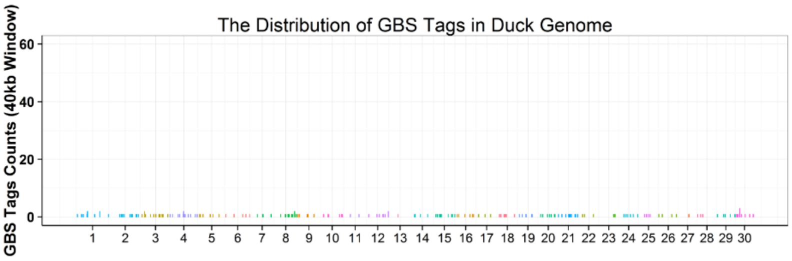

***Bmt*I**

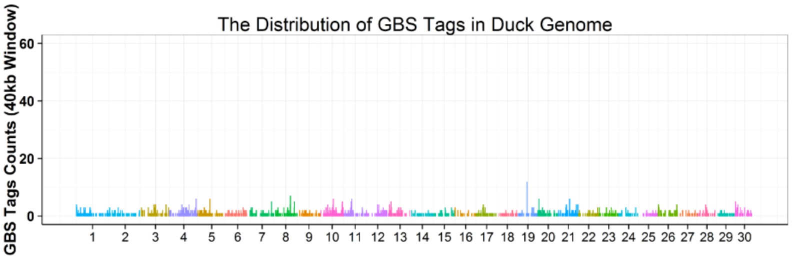

***Ban*I**

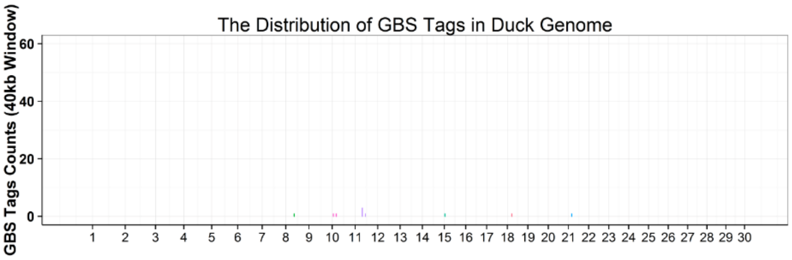

***Nar*I**

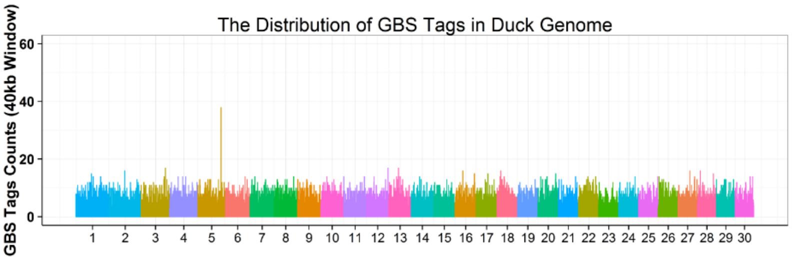

***Dpn*II**

Figure S2. Distribution of silico digested fragments count in 40-kb windows along the longest of the first thirty persudo-chromosome. Genomic sequences were distinguished by color.
